# Boston Letter

**Published:** 1902-09

**Authors:** 


### Domestic Correspondence.

### BOSTON LETTER.

TO THE EDITOR:

SIR,—The past few months have been of great importance to the dental societies of Massachusetts. With the coming of summer and the completion of school courses there is a culmination of dental efforts. The Harvard Odontological Society began its twenty-fifth year with Dr. Julius G. W. Werner occupying the presidential chair. That he fills it worthily goes without saying. His genial manner and his efforts to obtain every possible information from every source make the papers, and especially the discussions which follow, of great interest. Then the introduction of speakers from the medical profession has broadened the scope of the Society and opened paths of research which we ordinarily pass by with little notice.

Again, this Society has changed its constitution and by-laws so that its candidates for membership now must be graduates of two years' standing. The society was growing so rapidly that it was fast becoming unwieldy, and while there is no desire to check healthy growth, it was felt that by passing these measures a more sturdy if no less rapid increase would result. The Harvard Alumnæ held their annual meeting on the 23d of June. The exercises at the dental school were of even more interest than usual. We came and saw, and on every side there was still more to see, and so many were the attractions that it was hard to choose one and see it through, and thereby slight something else.

There was a large number of patients present showing work of every variety, and the degree of skill shown speaks well for the future of the profession. It was very interesting to notice how styles change in dentistry. Three or four years ago it was the fashion to band all porcelain crowns, but I noticed that there had been a general return to the Latimer type. And their superiority in suitable cases could not be doubted. There were the two crowns side by side, the one with purple, congested gums, the other clean and healthy. Sometimes we see in clinics that which astounds us, and that was certainly the case with myself when I saw the hydrochlorate of cocaine injected in amounts of one grain and upward. To be sure, the gentleman who gave this demonstration said that its toxic effect was overcome by the other ingredients, but I could not help wondering whether he really appreciated the missile he was using and its terrible effects in some cases. Nine hundred and ninety-nine cases might be all right, but is it worth the while if he should lose the thousandth?

The banquet in the evening was even too much of a success, as more came than there were seats for. This was due to the fact that many of the members did not reply to the secretary's notice. The Rev. Alexander McKenzie gave a very fine address, the custom being that the annual address shall be given by some one outside of the profession, and usually has no direct bearing upon professional matters. President Elliot was present, and spoke at some length. His remarks were received with great enthusiasm. The warm regard in which he is held and the close personal interest he has for the graduates was never more in evidence than during that evening. After listening to speeches by each of the professors of the school, the meeting was adjourned until the following year.

The meeting of the Massachusetts Dental Society at the Hotel

Brunswick drew a very large number of dentists to the city. The State society represents everybody, and consequently has a much larger field of influence than any other organization. Here also the dentist is brought in touch with the manufacturer, and the result is of the greatest benefit to both. Nowhere has the dentist a better opportunity to see what is being done elsewhere in the profession. And the practibility of the clinic is enforced by an opportunity to see the dealers' products and to obtain them, or to test their usefulness. One thing that impressed me was the presence of several agents for companies which manufacture specialties for the general practitioner. It was another link to show how the medical and dental professions are steadily drawing closer, so that co-operation is essential to both and is becoming more so every day. It is greatly to be regretted that these meetings cannot be held somewhere where there would be better opportunity for every one to see and understand the clinics. At present a favored few in front have a splendid chance to see, while the rest can only catch an occasional glimpse of the operator and patient, to say nothing of seeing what is being done. One way that this could be remedied would be to have circular portable rails so as to keep a fair space about the chair, and also small portable amphitheatres could be made, stored away when not in use, and brought out as the occasion requires.

The banquet in the evening was well attended, and when the hour of adjournment came the feeling was that a good deal had been accomplished gastronomically and otherwise, and that every one was entitled to a good summer's rest.

